# Supplementary material for: Neutrophil-to-Lymphocyte and Platelet-to-Lymphocyte Ratios as Prognostic Biomarkers in Unresectable Hepatocellular Carcinoma Treated with Atezolizumab plus Bevacizumab
Source: Cancers (Basel). 2022 Nov 26;14(23):5834. doi: 10.3390/cancers14235834 (PMC9737420; doi:10.3390/cancers14235834)
Supplement: Supplementary file 1 [file cancers-14-05834-s001.zip › cancers-2038166-supplementary.pdf]

cancers

MDPI

Supplementary Materials

Neutrophil-To-Lymphocyte and Platelet-to-Lymphocyte Ratios as Prognostic Biomarkers in Unresectable Hepatocellular Carcinoma Treated with Atezolizumab plus Bevacizumab

Yue Linda Wu, Claudia Angela Maria Fulgenzi, Antonio D'Alessio, Jaekyung Cheon, Naoshi Nishida, Anwaar Saeed, Brooke Wietharn, Antonella Cammarota, Tiziana Pressiani, Nicola Personeni, Matthias Pinter, Bernhard Scheiner, Lorenz Balcar, Yi-Hsiang Huang, Samuel Phen, Abdul Rafeh Naqash, Caterina Vivaldi, Francesca Salani, Gianluca Masi, Dominik Bettinger, Arndt Vogel, Martin Schönlein, Johann von Felden, Kornelius Schulze, Henning Wege, Peter R. Galle, Masatoshi Kudo, Lorenza Rimassa, Amit G. Singal, Rohini Sharma, Alessio Cortellini, Vincent E. Gaillard, Hong Jae Chon, David J. Pinato and Celina Ang

Table S1. Multivariate Cox Regression Analysis for Overall Survival Excluding PLR.

	HR; 95% CI	*P* value
AFP<br>≥400 vs. <400	1.49;0.98-2.27	0.06
ALBI<br>2 vs. 1	2.44;1.48-4.02	<0.001*
Child Pugh<br>6 vs. 5	1.42;0.91-2.23	0.120
NLR<br>≥5 vs. <5	1.95;1.21-3.15	0.006*
PVT<br>Yes vs. No	1.54;1.00- 2.36	0.049*
Previous LRT<br>Yes vs. No	0.87;0.64-1.18	0.377

* Statistically significant (P value < 0.05); PLR: platelet-to-lymphocyte ratio; HR: hazard ratio; CI: confidence interval; AFP: alpha fetoprotein; ALBI: albumin-bilirubin; NLR: neutrophil-to-lymphocyte ratio; PVT: portal vein thrombosis; LRT: locoregional therapy.

Table S2. Multivariate Cox Regression Analysis for Overall Survival Excluding NLR.

	HR; 95% CI	*P* value
**AFP**<br>≥400 vs. <400	1.43;0.93-2.20	0.099
**ALBI**<br>2 vs. 1	2.59;1.58-4.24	<0.001*
**Child Pugh**<br>6 vs. 5	1.53;0.98-2.39	0.059
**PLR**<br>≥300 vs. <300	1.48;0.80-2.74	0.211
**PVT**<br>Yes vs. No	1.56;1.02- 2.40	0.039*
**Previous LRT**<br>Yes vs. No	0.91;0.68-1.23	0.551

* Statistically significant (P value < 0.05); NLR: neutrophil-to-lymphocyte ratio; HR: hazard ratio; CI: confidence interval; AFP: alpha fetoprotein; ALBI: albumin-bilirubin; PLR: platelet-to-lymphocyte ratio; PVT: portal vein thrombosis; LRT: locoregional therapy.

Likelihood ratio test: < 0.001* (first model, Supplemental Table 1, including NLR is superior)

Table S3. Multivariate Cox Regression Analysis for Progression-Free Survival Excluding PLR.

	**HR; 95% CI**	***P* value**
**AFP**<br>≥400 vs. <400	1.42;1.04-1.95	0.027*
**ALBI**<br>2 vs. 1	1.41;1.03-1.93	0.030*
**NLR**<br>≥5 vs. <5	1.38;0.94-2.04	0.102

* Statistically significant (P value < 0.05); PLR: platelet-to-lymphocyte ratio; HR: hazard ratio; CI: confidence interval; AFP: alpha fetoprotein; ALBI: albumin-bilirubin; NLR: neutrophil-to-lymphocyte ratio.

Table S4. Multivariate Cox Regression Analysis for Progression-Free Survival Excluding NLR.

	**HR; 95% CI**	***P* value**
**AFP**<br>≥400 vs. <400	1.40;1.02-1.93	0.038*
**ALBI**<br>2 vs. 1	1.43;1.05-1.95	0.025*
**PLR**<br>≥300 vs. <300	1.40;0.83-2.35	0.205

* Statistically significant (P value < 0.05); NLR: neutrophil-to-lymphocyte ratio; HR: hazard ratio; CI: confidence interval; AFP: alpha fetoprotein; ALBI: albumin-bilirubin; PLR: platelet-to-lymphocyte ratio.

Likelihood ratio test: < 0.001* (first model, Supplemental Table 3, including NLR is superior)
